# Comparative Analysis of Periodontal Pain According to the Type of Precision Orthodontic Appliances: Vestibular, Lingual and Aligners. A Prospective Clinical Study

**DOI:** 10.3390/biology10050379

**Published:** 2021-04-28

**Authors:** Laura Antonio-Zancajo, Javier Montero, Daniele Garcovich, Mario Alvarado-Lorenzo, Alberto Albaladejo, Alfonso Alvarado-Lorenzo

**Affiliations:** 1 Faculty of Medicine, University of Salamanca, Avenida Alfonso X el Sabio s/n, 37007 Salamanca, Spain; javimont@usal.es (J.M.); albertoalbaladejo@usal.es (A.A.); kuki@usal.es (A.A.-L.); 2 Department of Dentistry, European University of Valencia, Passeig de lÁlbereda, 7, 46010 Valencia, Spain; daniele.garcovich@universidadeuropea.es; 3 Department of Dentistry, European University Miguel de Cervantes, Padre Julio Chevalier, 2, 47012 Valladolid, Spain; maalvarado@uemc.es

**Keywords:** orthodontics, pain, location, degree of pain, low-friction brackets, lingual orthodontics, Invisalign, aligners

## Abstract

**Simple Summary:**

Currently, there are new trends in orthodontics that are increasingly being used in clinical practice, such as aligners. It is important to know the influence of pain perception in orthodontic patients in order to be able to apply less painful and more comfortable techniques for patients. The advancement of different fixed and removable appliances, using precision digital fabrication methods, is increasingly harnessed in the daily life of our society. Negative experiences such as periodontal pain can cause the rejection of orthodontic treatment. Therefore, the study of the location, intensity and type of pain according to the different techniques is beneficial for improving the quality and success of orthodontic treatment.

**Abstract:**

The objective of this prospective clinical study was to analyze the pain (intensity, location and type) that patients presented after the placement of different types of orthodontic appliances: conventional, low friction, lingual and aligners. The sample consisted of 120 patients divided into four groups: conventional (CON), low friction (LF), lingual (LO) and aligners (INV). The participants were given the Short-Form McGill Pain Questionnaire (Ortho-SF-MPQ), where they had to record the pain intensity (no pain, mild, moderate or intense) and the periodontal location at different time points, from the first 4 h to 7 days after the start of treatment. In all the study groups, the most frequent location was both anterior arches, with maximum values between 56.7% (CON group at 24 h) and 30% (LO group at 4 h). The “whole mouth” and “complete lower arch” locations were indicated only by the patients in the lingual group. Regarding pain intensity, the patients reported a higher percentage of mild–moderate pain during the first 3 days of treatment (96.7% in LO at 4 h, 86.7% in CON, 83.3% in LF and 90% in INV at 24 h); later, the reported pain decreased to no pain/mild pain, especially in the lingual group, until reaching values close to zero at 7 days post-treatment. The most frequent type of pain was acute in the low friction and lingual groups (with maxima of 60% and 46.7% at 24 h, respectively). On the contrary, in the conventional (36.7% at 4 h) and Invisalign (40% at 24 h) groups, the sensitive type was the most frequent. There are differences regarding periodontal pain in its intensity, location and type according to the use of different orthodontic techniques.

## 1. Introduction

There are new orthodontic techniques, such as aligners, that should be compared with conventional techniques. According to patients, dental treatments are associated with suffering a certain degree of pain. We found that 77% of patients suffered pain during visits to orthodontists [1] and 6% of patients even stated that dental treatment has always been painful [2]. Suffering pain in a previous visit to the dentist causes the pain threshold to decrease in subsequent visits [3,4]. In orthodontic treatment, around 90% of patients affirm that pain and discomfort were the main sources of inconvenience and could even act as an obstacle to starting treatment and/or cause delays. These results are independent of the technique used. Normally, the pain that patients experience with orthodontic treatment is usually mild/moderate and of a short duration [5,6,7,8,9,10,11,12]. Most authors find that the pain peak occurs at 24 h and decreases to values close to zero after 7 days of treatment [13,14,15]. Although the degree of pain experienced will be perceived differently between individuals, some patients consider orthodontic pain to be greater in incidence and severity than the pain experienced during tooth extractions [16,17,18]. For some authors, this pain manifests itself in a general dentogingival manner in 65.7% of cases and 39% as localized [19,20]. Regarding the type of pain, it is usually localized during the chewing function, and is not usually spontaneous [21,22,23]. 

Patients describe the sensation of pain and discomfort as pressure, tension, pain in the teeth and general pain [6]. If we apply intense initial forces in our treatment, we produce a certain level of pain. According to different authors, the application of a higher degree of force seems to increase the degree of biological and inflammatory responses. Large forces will cause greater periodontal compression and, therefore, greater pain [10,24]. In his 2009 study, Ogura reported that chewing pain was greater when the applied forces were greater; however, there were no major differences for spontaneous pain [25]. Gentle movements with light, continuous forces would produce less discomfort to patients [10,26]. For other authors, a direct relationship between the level of pain and the amount of crowding and magnitude of force applied has not been demonstrated [22]. 

Most of the authors consulted consider that the pain experienced by patients and the discomfort associated with orthodontic treatment will have a negative influence on the quality of their oral life [4,26,27,28,29]. The orthodontic technique used also seems to influence the level of pain and the quality of life that patients experience during treatment [13].

In the literature, we find that most of the studies that analyze the location of pain with the different techniques do so by comparing pain in the mucosa, lips and tongue. The results show that there would be greater lingual pain in lingual orthodontics and greater labial and vestibular pain in conventional vestibular orthodontics [30,31]. Wu et al. also stated in their study that there were no differences in pain between both techniques, but there were differences in their location [31]. 

For measuring pain, the McGill Pain Questionnaire (MPQ) can be used, since it measures the three dimensions of pain according to Melzack: sensory, affective and evaluative [32]. Our study used the Short-Form McGill Pain Questionnaire (Ortho-SF-MPQ), which was previously validated in patients with orthodontics by Sandhu [33]. This study analyzed the intensity, location and type of pain suffered at the periodontal level by patients after the placement of different types of orthodontic appliances during the first phase of treatment. 

The importance of this study lies in the possibility of comparatively assessing the characteristics of pain that our patients present after the placement of orthodontic appliances at the level of periodontal pain. Knowing if there are differences in intensity and type of pain would allow us to gain another point of consideration when choosing the type of appliance to be placed. The increased demand for aligners in recent decades has led to their comparison with the latest vestibular and lingual bracket systems, together with the use of high-precision digital tools. Therefore, they were analyzed in the present clinical study in reference to perception of periodontal pain.

The null hypothesis of our study is that there are no differences in periodontal pain in terms of location, intensity and type in patients with different orthodontic techniques.

## 2. Materials and Methods 

A clinical study was carried out to analyze pain perception in patients with fixed and removable orthodontics. All the participants were informed about the protocols and agreed to participate voluntarily. The participants chose and knew the type of equipment with which they were going to be treated. In total, there were 120 participants, of which 55% were women (66) and 45% men (44). None of the participants who consented to the study withdrew during its development. This work followed the guidelines established by the Declaration of Helsinki for human research; the project was approved by the bioethics committee of the University of Salamanca (USAL_16/060) and was carried out during the first semester of 2020.

This prospective clinical study was carried out on 120 patients, a sample size similar to other published studies [14,15]. To determine the sample size, previous studies were used as an initial guide; however, it was ultimately calculated with the online tool Raosoft (Raosoft Inc., Seattle, WA, USA). A margin of error of 5% and a confidence level of 95% were applied.

The sample was divided into four study groups of 30 people each.

Group 1: Vestibular conventional brackets—CON (Victory Series^®^, 3M, Rogers, AK, USA).

Group 2: Vestibular low friction brackets—LF (Synergy^®^, Rocky Mountain Orthodontics, Denver, CO, USA).

Group 3: Lingual brackets—LO (STB^®^ from Ormco, Orange, CA, USA).

Group 4: Aligners—INV (Invisalign^®^ from Align Technology Inc., San Jose, CA, USA).

In the first three groups, the bracket slot size was 018” × 0.025”. A 0.014” Cooper NiTI archwire was used in the vestibular groups and a 0.013” universal preform Cooper NiTI archwire was used in the lingual group according to the STB^®^ technique protocol defined by Dr. Scuzzo [34]. All of them were joined by 0.10-inch metal wires. 

For the selection of the participants, the following inclusion criteria were followed: patients between 18 and 40 years of age with permanent dentition and skeletal class I or II and mild III (Ricketts convexity 2 mm ± 2); with a negative tooth size–arch length discrepancy (TSALD) between −6 and −2 mm [35]; those who had not had any tooth removed (except third molars) and who had not received prior orthodontic treatment. They had to have good oral (dental and periodontal) and general health (no previous systemic diseases). The patients who started the study had no periodontal pathology. However, after the placement of the orthodontic appliances, they were given oral hygiene guidelines for proper periodontal maintenance [36]. As exclusion criteria, the patients did not: suffer severe malformations, need orthodontic surgical treatment, take medication (analgesics, antidepressants and/or anticonvulsants), nor did they have any medical condition that influenced the perception of pain, and the lingual anatomy of their teeth allowed for correct cementation of the brackets in the case of lingual orthodontic patients. 

After the placement of the appliances, the patients received a Short-Form McGill Pain Questionnaire (Ortho-SF-MPQ), consisting of a drawing of the two dental arches where they had to highlight the location of the pain suffered and a questionnaire where they were to fill in the intensity (no pain, mild, moderate or severe) and the type of pain (throbbing, shooting, stabbing, burning, acute, piercing, cramping, dull, heavy, sensitive, exhausting, cruel, terrible and frightening). For a better understanding of the results regarding the type of pain, those with a percentage higher than 15% were considered (these types of pain were acute, dull, sensitive, throbbing, and stabbing). The remaining types of pain, due to their low individual incidence, were considered as a single group labeled “other types of pain” [37,38]. 

Both questionnaires were clearly explained to the participants before starting the treatment to ensure they completely understood them, and they were asked to fill them in at different times after the placement of the appliance: 4 h (T4h), 8 h (T8h), 24 h (T1), 2 days (T2), 3 days (T3), 4 days (T4), 5 days (T5), 6 days (T6) and 7 days (T7) [22,39]. 

For analysis of the location, according to the areas marked by the patients (one or several) and to delineate them better it was decided to divide them into upper arch (anterior and/or posterior), lower arch (anterior and/or posterior), pain in both arches (anterior and/or posterior), pain in the entire mouth and pain in the entire lower arch. 

To analyze the data, we used the SPSS v. 20 software (SPSS Inc., Chicago, IL, USA). Mean and standard deviation were used to describe the distribution of the quantitative variables of the studied population. For the nominal and ordinal data, the sample distribution (the number of patients and the corresponding percentage) was determined. To compare two or more nominal or ordinal distributions, the Chi-square test was performed. Two statistical significance levels were established *p* < 0.05, as statistically significant and *p* < 0.01 as highly significant.

## 3. Results

### 3.1. Characteristics of the Participants

After analysis of the 120 participants, it was found that the sample’s mean age was 30.0 ± 7.5 years. The degree of crowding presented by the patients was also analyzed prior to treatment, finding no statistically significant differences between the groups (Table 1).

### 3.2. Analysis of the Periodontal Location of Pain

Regarding the location of dental pain and the hours analyzed, we found statistically significant differences at 8 h and 4 days of analysis (*p* < 0.05), and at 4 h, 24 h, 2 days, 3 days, 5 days, 6 days and 7 days (*p* < 0.01) (Table 2).

The most frequent location reported by all the patients was “both anterior arches”. The maximum value was found at 4 h in the LO (30%) and INV (53.3%) groups and at 24 h in CON (56.7%) and LF (50%). The next most frequent locations of pain were “maxillary anterior” and “mandibular anterior” (Table 2).

We found that the types of pain “whole mouth” and “complete lower arch” were only referenced by lingual orthodontic patients with a maxima of 13.3% at 24 h and 16.7% at 8 h, respectively.

#### 3.2.1. Evolution of Pain in Both Anterior Arches

**Figure 1:**
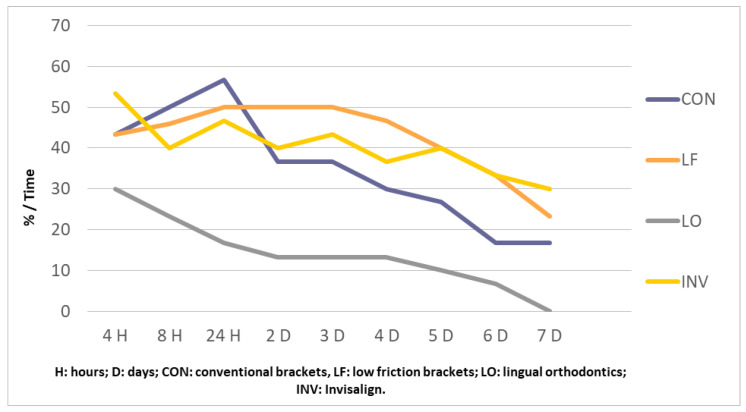
Evolution of both anterior arch pain (%).

In Figure 1, for both anterior arches, we can see that, in the LO group, the percentage of patients with pain was lower than that in the other three groups analyzed. The decrease in patients with pain in the LO group occured gradually from 4 h of treatment to 7 days. However, in the other three groups, there was an increase in patients with pain in this location at 24 h and the decrease showed more fluctuation during the first seven days of treatment.

#### 3.2.2. Evolution of Pain in the Anterior Maxillary Location 

**Figure 2:**
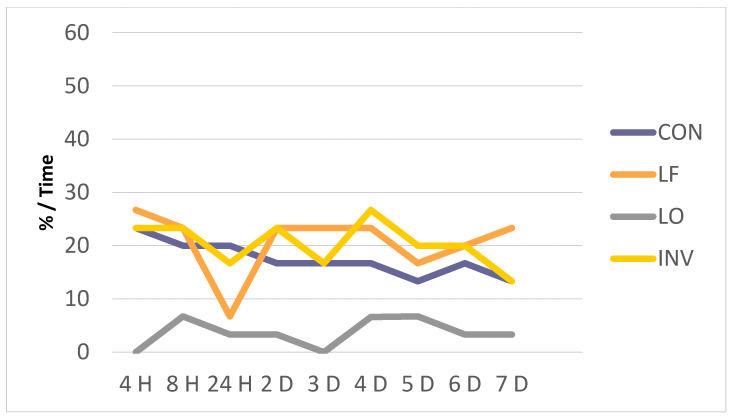
Evolution of anterior maxillary pain (%).

As in the previous case, we found that, at the level of the anterior maxilla, the LO group presented a lower percentage of patients with pain in this location than the other three groups, although with less difference. The LF group suffered a decrease at 24 h and an increase at 7 days in contrast to the CON and INV groups, where the number of patients with pain increased in this location at 24 h and decreased after 7 days of treatment (Figure 2).

#### 3.2.3. Evolution of Pain in the Anterior Mandibular Location

**Figure 3:**
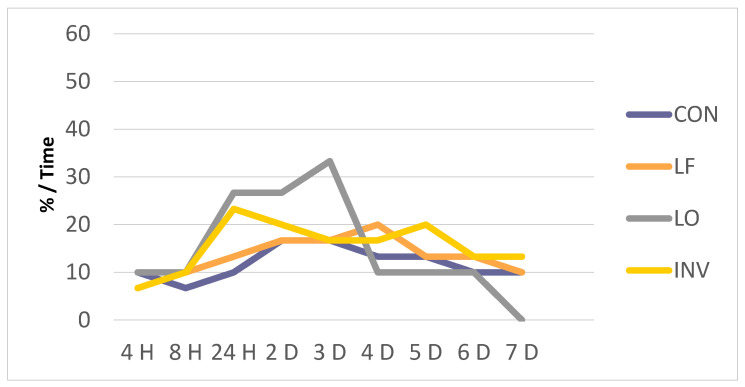
Evolution of anterior mandibular pain (%).

In Figure 3, presenting the anterior mandibular location, we observe that the number of patients with pain in all the groups increased in the first 24 h. In the LO group, this increase continued to occur up to 3 days after, with a maximum of 33.3%. This group had the highest percentage of patients with pain in this location between 24 h and 3 days; later, this group presents a sharp decrease, with values of 10% at 4 days, remaining until 6 days. Seven days after treatment, no patient presented pain in this location in the LO group compared to 10% of CON and LO, and 13.3% of INV (Figure 3).

### 3.3. Analysis of the Degree of Pain by Groups 

We found statistically significant differences in the degree of pain at T4h (*p* < 0.05) and between T3 and T7 (*p* < 0.01). However, we did not find statistically significant differences at 8 h and 2 days after the placement of the appliances (Table 3). 

In the first 4 h after treatment, conventional orthodontic patients (CON) rated their pain as mild (46.7%) and moderate (40.0%), compared to a mild intensity in the other three study groups (60.0% in LF and LO and 66.7% in INV). The level of pain increased in all groups to mild/moderate up to the fourth day of analysis. After 5 days of analysis, the percentage of patients without pain in the lingual orthodontic group was significantly higher than that in the other three treatment groups (Table 3). 

### 3.4. Analysis of the Type of Pain by Groups

In the analysis of the type of pain, we found statistically significant differences at all time points with a *p* < 0.01. In the first 24 h of analysis, “acute pain” was the most frequent type of pain in the low friction groups and in lingual orthodontics, followed by “sensitive pain”. In contrast, in the conventional and Invisalign groups, the most frequent type of pain referred to by patients was “sensitive pain”. Subsequently, these groups reported a more “stabbing and throbbing” pain in the case of the conventional group, and a type of “acute pain” in the Invisalign group (Table 4).

After 3 days and until the end of our analysis at 7 days, the number of patients with pain decreased, especially in the lingual orthodontic group. We observed how, in this group, the patients who still had pain mostly classified it as “acute” and “sensitive”, as did the patients in the Invisalign group. In conventional and low-friction cases, the types of pain referenced continue to be divided into several categories (Table 4).

## 4. Discussion

This study analyzed and compared the intensity, location, and type of pain with different types of appliances during the first phase of treatment. Most of the studies found in the literature analyze pain in conventional orthodontics [14,15,29]. There are few studies comparing more than two different orthodontic techniques [13,40].

During orthodontic treatment, different forces are applied through the brackets and arches, which cause tooth movement in the alveolar bone [9]. For some authors, forces of 20–40 g are enough to cause pain [41] and a greater degree of force will cause an increase in pain [10,24]. This degree of force and pain is related to the degree of crowding that patients present: the more crowding that occurs, the more force is released by the arch on the teeth as they regain their shape [10,24,42]. In our study, we have used a homogeneous sample with no statistically significant differences regarding osseodental discrepancy (Table 1). 

On the other hand, according to the literature analyzed, there are no differences in pain presented by patients with different arches used or sequences of arches [43,44,45]. In this study, the same arch section was used in the bracket techniques (CON, LF and LO groups) and the same bracket slot (0.018″).

In our study, we analyzed the difference in location at the periodontal level. We found that the most frequent location of pain in all the study groups was in both anterior arches, followed by the anterior maxillary and anterior mandibular pain (Table 2). Only the lingual orthodontic group reported pain throughout the mouth and in the entire lower arch. For some authors, there is a higher percentage of patients with pain in the anterior teeth than in posterior teeth with conventional vestibular fixed orthodontics, coinciding with our results [46,47,48] and more in the mandible than in the maxilla [49]. We consider that this greater pain at the anterior level may be related to the fact that the anterior teeth move more during the alignment phases (beginning of the treatment), they have smaller roots and, generally, it is where the highest degree of crowding is located. Other authors refer to dentogingival pain with fixed orthodontics; however, they do not specify its location [19]. 

Most of the authors reported that the degree of pain with orthodontic appliances during the first week of treatment was mild–moderate [9,10,11,12,23]. We obtained similar results in our study, where the pain is mild–moderate in the initial timepoints of analysis (up to two days) (Table 3). Subsequently, we found that the percentage of patients with no pain/mild pain in the lingual orthodontic group increased and was comparatively higher than the rest of the groups that continued to move between mild/moderate pain. At first, we found that it was the conventional and low-friction orthodontic patients who rated their pain as the most intense, even reaching 26.7% at 24 h in the low-friction group. According to the systematic review carried out by Long et al. in 2013, the degree of pain that occurs in lingual orthodontic and conventional orthodontic treatments is similar [50]. In a randomized clinical trial recently published by Casteluci et al., where they compared the intensity of pain between fixed appliances and aligners, they concluded that it was similar and mild–moderate with both techniques [51]. The same happens between conventional fixed orthodontics and low friction [52]. Similar results were obtained in our study with lingual orthodontics and aligners. Most of the authors consulted reported that there was less pain in treatment with aligners compared to treatments with fixed appliances, which agrees with our results [53,54,55,56]. In neither of the trials were lingual orthodontics analyzed in a comparative technique.

One of the limitations of this study was obtaining homogeneous samples with respect to sex and age, since both variants can influence the choice of treatment: adult and female patients choose more aesthetic techniques such as lingual orthodontics and aligners. Some authors found no differences in pain intensity and age or sex of the patients in adult patients [14,26,46,57,58]. We can also add that this was a short-term study, the reason being that the first week was when patients felt the greatest pain and it decreased as months passed during orthodontic treatment [51]. Long-term studies are needed to see the different behavior that different orthodontic techniques may have in reference to periodontal pain.

In the literature, we found another study that compared four different orthodontic techniques [13] with respect to the level of pain and the influence on the quality of life. This study, analyzing the same techniques, focuses on the study of the degree of pain and its location at the periodontal level. In order to improve our limitations, in future studies, we intend to increase the sample size and the analysis time, as well as to improve and equalize the percentage of women and men assessed, including innovative topics both in this line of research and in other future ones [59].

## 5. Conclusions

After analyzing the results, we found that the most frequently affected dental location was both anterior arches, followed by the “anterior maxilla” and mandible, in the four study groups. Regarding the degree of pain, it was mild/moderate in the conventional group and mild in the other three groups in the first 24 h of the study, progressively decreasing with greater speed in the lingual orthodontic patients. In the first 24 h, the most frequent pain was acute pain in the BF and LO groups and sensitive pain in CON and INV. Treatment with aligners (Invisalign) behaved similarly to the rest of the techniques in terms of location and degree of pain.

## Figures and Tables

**Table 1:** Demographic characteristics of participants (*n* = 120).

	Age (Years)		Sex				TSALD			
	Mean	SD	Men		Woman		Upper		Lower	
			N	%	N	%	Mean	SD.	Mean	SD.
Conventional Brackets (CON) (n = 30)	24.7	4.1	13	43.3	17	56.7	−3.1	1.0	−3.3	1.3
Low-Friction Brackets (LF) (n = 30)	28	9.7	12	40	18	60	−3.1	0.7	−2.7	1.2
Lingual Brackets (LO) (n = 30)	33.8	8.2	13	43.3	17	56.7	−3.0	1.6	−3.4	1.5
Invisalign (INV) (n = 30)	33.4	5.1	16	53.3	14	46.6	−2.6	1.6	−2.6	1.7

TSALD: tooth size–arch length discrepancy.

**Table 2:** Description of dental pain between groups (*n* = 120).

Time	Groups	No Pain		Both Arches				Mandible				Maxilla				Whole Mouth		Lower Arch	
				Anterior		Posterior		Anterior		Posterior		Anterior		Posterior					
		N	%	N	%	N	%	N	%	N	%	N	%	N	%	N	%	N	%
T4h (4 h) **	CON	0	0.0	13	43.3	3	10.0	3	10.0	2	6.7	7	23.3	2	6.7	0	0.0	0	0.0
	LF	5	16.7	13	43.3	0	0.0	2	6.7	1	3.3	8	26.7	1	3.3	0	0.0	0	0.0
	LO	8	26.7	9	30.0	4	13.3	3	10.0	0	0.0	0	0.0	1	3.3	0	0.0	0	0.0
	INV	1	3.3	16	53.3	4	13.3	2	6.7	0	0.0	7	23.3	0	0.0	0	0.0	0	0.0
Chi: 46.09, fd: 24; *p*-value: 0.004																			
T8h (8 h) *	CON	0	0.0	15	50.0	3	10.0	2	6.7	2	6.7	6	20.0	2	6.7	0	0.0	0	0.0
	LF	3	10.0	14	46.7	1	3.3	3	10.0	1	3.3	7	23.3	1	3.3	0	0.0	0	0.0
	LO	3	10.0	7	23.3	4	13.3	3	10.0	2	6.7	2	6.7	1	3.3	3	10.0	5	16.7
	INV	1	3.3	12	40.0	3	10.0	6	20.0	0	0.0	7	23.3	1	3.3	0	0.0	0	0.0
Chi: 41.21; fd: 24; *p*-value: 0.016																			
T1 (24 h) **	CON	0	0.0	17	56.7	1	3.3	3	10.0	1	3.3	6	20.0	2	6.7	0	0.0	0	0.0
	LF	0	0.0	15	50.0	2	6.7	4	13.3	1	3.3	8	6.7	0	0.0	0	0.0	0	0.0
	LO	4	13.3	5	16.7	2	6.7	8	26.7	2	6.7	1	3.3	1	3.3	4	13.3	3	10.0
	INV	0	0.0	14	46.7	3	10.0	7	23.3	1	3.3	5	16.7	0	0.0	0	0.0	0	0.0
Chi: 53.21; fd: 24; *p*-value: 0.001																			
T2 (2 days) **	CON	1	3.3	11	36.7	4	13.3	5	16.7	2	6.7	5	16.7	2	6.7	0	0.0	0	0.0
	LF	1	3.3	15	50.0	1	3.3	5	16.7	1	3.3	7	23.2	0	0.0	0	0.0	0	0.0
	LO	7	23.3	4	13.3	2	6.7	8	26.7	1	3.3	1	3.3	1	3.3	3	10.0	3	10.0
	INV	2	6.7	12	40.0	3	10.0	6	20.0	0	0.0	7	23.3	0	0.0	0	0.0	0	0..0
Chi: 53.73; fd: 24; *p*-value: 0.00																			
T3 (3 days) **	CON	1	3.3	11	36.7	4	13.3	5	16.7	2	6.7	5	16.7	2	6.7	0	0,0	0	0,0
	LF	1	3.3	15	50.0	1	3.3	5	16.7	1	3.3	7	23.3	0	0.0	0	0,0	0	0,0
	LO	9	30.0	4	13.3	3	10.0	10	33.3	0	0.0	0	0.0	0	0.0	1	3.3	3	10.0
	INV	3	10.0	13	43.3	3	10.0	5	16.7	1	3.3	5	16.7	0	0.0	0	0,0	0	0,0
Chi: 49.7; fd: 24; *p*-value: 0.002																			
T4 (4 days) **	CON	5	16.7	9	30.0	4	13.3	4	13.3	1	3.3	5	16.7	2	6.7	0	0.0	0	0.0
	LF	2	6.7	14	46.7	0	0.0	6	20.0	1	3.3	7	23.3	0	0.0	0	0.0	0	0.0
	LO	16	53.3	4	13.3	1.	3.3	3	10.0	0	0.0	2	6.7	0	0.0	0	0.0	4	13.3
	INV	3	10.0	11	36.7	1	3.3	5	16.7	2	6.7	8	26.7	0	0.0	0	0,0	0	0.0
Chi: 55.74; fd: 21; *p*-value: 0.00																			
T5 (5 days) **	CON	8	26.7	8	26.7	4	13.3	4	13.3	2	6.7	4	13.3	0	0.0	0	0.0	0	0.0
	LF	7	23.3	12	40.0	0	0.0	4	13.3	1	3.3	5	16.7	1	3.3	0	0.0	0	0.0
	LO	19	63.3	3	10.0	0	0.0	3	10.0	0	0.0	2	6.7	0	0.0	0	0.0	3	10.0
	INV	3	10.0	12	40.0	1	3.3	6	20.0	2	6.7	6	20.0	0	0.0	0	0.0	0	0.0
Chi: 47.45; fd: 21; *p*-value: 0.001																			
T6 (6 days) **	CON	12	40.0	5	16.7	3	10.0	3	10.0	2	6.7	5	16.7	0	0.0	0	0,0	0	0,0
	LF	8	26.7	10	33.3	0	0.0	4	13.3	1	3.3	6	20.0	1	3.3	0	0.0	0	0.0
	LO	24	80.0	2	6.7	0	0.0	3	10.0	0	0.0	1	3.3	0	0.0	0	0.0	0	0.0
	INV	9	30.0	10	33.3	0	0.0	4	13.3	0	0.0	6	20.0	1	3.3	0	0.0	0	0.0
Chi: 37.94; fd: 18; *p*-value: 0.004																			
T7 (7 days) **	CON	14	46.7	5	16.7	2	6.7	3	10.0	1	3.3	4	13.3	1	3.3	0	0.0	0	0.0
	LF	11	36.7	7	23.3	0	0.0	3	10.0	1	3.3	7	23.3	1	3.3	0	0.0	0	0.0
	LO	29	96.7	0	0.0	0	0.0	0	0.0	0	0.0	1	3.3	0	0.0	0	0.0	0	0.0
	INV	12	40.0	9	30.0	0	0.0	4	13.3	0	0.0	4	13.3	1	3.3	0	0.0	0	0.0
Chi: 38.53; fd: 18; *p*-value: 0.003																			

* Statistically significant results (*p <* 0.05); ** statistically significant results (*p <* 0.01); CON: conventional brackets group (*n* = 30); LF: low friction bracket group (*n* = 30); LO: lingual brackets group (*n* = 30); INV: Invisalign aligners group (*n* = 30).

**Table 3:** Comparison of ordinal pain by groups (*n* = 120).

Time	Pain Intensity	Conventional (*n* = 30)		Low Friction (*n* = 30)		Lingual (*n* = 30)		Invisalign (*n* = 30)	
T4h *		N	%	N	%	N	%	N	%
	No pain	0	0.0	6	20.0	7	23.3	1	3.3
	Mild	14	46.7	18	60.0	18	60.0	20	66.7
	Moderate	12	40.0	4	13.3	4	13.3	8	26.7
	Intense	4	13.3	2	6.7	1	3.3	1	3.3
Chi: 20.94; fd: 9; *p*-value: 0.013									
T8h	No pain	0	0.0	3	10.0	1	3.3	1	3.3
	Mild	8	26.7	16	53.3	13	43.3	16	53.3
	Moderate	17	57.7	9	30.0	14	46.7	11	36.7
	Intense	5	16.7	2	6.7	2	6.7	2	6.7
Chi: 12.36; fd: 9; *p*-value: 0.19									
T24h	No pain	0	0.0	0	0.0	0	0.0	0	0.0
	Mild	8	26.7	12	40.0	11	36.7	11	36.7
	Moderate	18	60.0	10	33.3	18	60.0	16	53.3
	Intense	4	13.3	8	26.7	1	3.3	3	10.0
Chi: 10.13; fd: 6; *p*-value: 0.12									
T2	No pain	0	0.0	0	0.0	4	13.3	2	6.7
	Mild	13	43.3	10	33.3	14	46.7	12	40.0
	Moderate	12	40.0	13	43.3	11	36.7	14	46.7
	Intense	5	16.7	7	23.3	1	3.3	2	6.7
Chi: 14.55; fd: 9; *p*-value: 0.11									
T3 **	No pain	1	3.3	3	3.3	10	3.3	3	10.0
	Mild	16	53.3	18	60.0	17	56.7	14	46.7
	Moderate	9	30.0	6	20.0	3	10	11	36.7
	Intense	4	13.3	5	16.7	0	0.0	2	6.7
Chi: 25.57; fd: 9; *p*-value: 0.002									
T4 **	No pain	5	16.7	2	6.7	14	46.7	3	10.0
	Mild	15	50.0	18	60.0	15	50.0	22	73.3
	Moderate	6	20.0	8	26.7	1	3.3	4	13.3
	Intense	4	13.3	1	3.3	0	0.0	1	3.3
Chi: 31.52; fd: 12; *p*-value: 0.002									
T5 **	No pain	7	23.3	7	23.3	19	63.3	3	10.0
	Mild	16	53.3	18	60.0	11	36.7	23	76.7
	Moderate	17	23.3	5	16.7	0	0.0	0	0.0
	Intense	0	0.0	0	0.0	0	0.0	0	0.0
Chi: 26.85; fd: 6; *p*-valor: 0.00									
T6 **	No pain	11	36.7	8	26.7	24	83.3	10	33.3
	Mild	13	43.3	19	63.3	5	16.7	19	63.3
	Moderate	6	20.0	3	10	0	0.0	1	3.3
	Intense	0	0.0	0	0.0	0	0.0	0	0.0
Chi: 31.24; fd: 6; *p*-value: 0.00									
T7 **	No pain	13	43.3	11	36.7	27	90.0	13	43.3
	Mild	14	46.7	18	60.0	3	10.0	16	53.3
	Moderate	3	10	1	3.3	0	0.0	1	3.3
	Intense	0	0.0	0	0.0	0	0.0	0	0.0
Chi: 24.62; fd: 6; *p*-value: 0.00									

* Statistically significant results (*p* < 0.05); ** statistically significant results (*p* < 0.01).

**Table 4:** Comparison of type of pain by groups (*n* = 120).

Time	Type of Pain	Conventional (*n* = 30)		Low Friction (*n* = 30)		Lingual (*n* = 30)		Invisalign (*n* = 30)	
T4h **		N	%	N	%	N	%	N	%
	No pain	1	3.3	6	20,0	7	23.3	1	3.3
	Acute	2 ^A^	6.7	8 ^A,C^	26.7	13 ^C^	43.3	8 ^A,C^	26.7
	Dull	1	3.3	0	0.0	0	0.0	0	0.0
	Sensitive	11 ^A,D^	36.7	3 ^A^	10.0	8 ^A,D^	26.7	13 ^D^	43.3
	Throbbing	6	20.0	4	13.3	1	3.3	4	13.3
	Stabbing	5	16.7	5	16.7	0	0.0	0	0.0
	Other types of pain	4	13.3	4	13.3	1	3.3	4	13.3
Chi: 49.16; fd: 27; *p*-value: 0.006									
T8h **	No pain	0	0.0	3	10.0	1	3.3	0	0.0
	Acute	4	13.3	12	40.0	13	43.3	7	23.3
	Dull	1	3.3	2	6.7	1	3.3	0	0.0
	Sensitive	11 ^B,D^	36.7	3 ^B^	10.0	10 ^B,D^	33.3	14 ^D^	46.7
	Throbbing	6	20.0	2	6.7	2	6.7	6	20.0
	Stabbing	6	20.0	5	16.7	0	0.0	0	0.0
	Other types of pain	2	6.7	3	10.0	3	10.0	3	10.0
Chi: 44.26; fd: 24; *p*-value: 0.007									
T24h **	No pain	0	0.0	0	0.0	0	0.0	0	0.0
	Acute	4 ^A^	13.3	18^C^	60.0	14 ^C^	46.7	10 ^A,C^	33.3
	Dull	1	3.3	3	10.0	1	3.3	2	6.7
	Sensitive	9 ^C^	30.0	0 ^B^	0.0	12 ^C^	40.0	11 ^C^	36.7
	Throbbing	8	26.7	1	3.3	2	6.7	4	13.3
	Stabbing	5	16.7	4	13.3	0	0.0	1	3.3
	Other types of pain	3	10.0	4	13.3	1	3.3	2	6.7
Chi: 48.59; fd: 24; *p*-value: 0.002									
T2 **	No pain	0	0.0	0	0.0	4	13.3	2	6.7
	Acute	3 ^A^	10.0	17 ^B^	56.7	10 ^A,B^	33.3	13 ^B^	43.3
	Dull	1	3.3	3	10.0	0	0.0	0	0.0
	Sensitive	8 ^B,C^	26.7	1 ^B^	3.3	13 ^C^	43.3	10 ^C^	33.3
	Throbbing	11 ^A^	36.7	1 ^C^	3.3	1 ^C^	3.3	4 ^A,C^	13.3
	Stabbing	4	13.3	4	13.3	1	3.3	0	0.0
	Other types of pain	3	10.0	4	13.3	1	3.3	1	3.3
Chi: 63.87; fd: 27; *p*-value: 0.000									
T3 **	No pain	1	3.3	1	3.3	10	33.3	3	10.0
	Acute	3	10.0	12	40.0	10	33.3	12	40.0
	Dull	1	3.3	9	30.0	0	0.0	0	0.0
	Sensitive	8	26.7	1	3.3	8	26.7	10	33.3
	Throbbing	9	30.0	0	0.0	1	3.3	3	10.0
	Stabbing	4	13.3	3	10.0	0	0.0	1	3.3
	Other types of pain	4	13.3	4	13.3	1	3.3	1	3.3
Chi: 89.91; fd: 30; *p*-value: 0.000									
T4 **	No pain	5 ^A^	16.7	2 ^A^	6.7	14 ^C^	46.7	3 ^A,C^	10.0
	Acute	2 ^A^	6.7	10 ^B^	33.3	7 ^A,B^	23.3	12 ^B^	40.0
	Dull	1 ^A^	3.3	9 ^B^	30.0	0 ^A^	0.0	0 ^A^	0.0
	Sensitive	8 ^B,D^	26.7	3 ^B^	10.0	7 ^B,D^	23.3	11 ^D^	36.7
	Throbbing	8 ^A^	26.7	0 ^C^	0.0	1 ^C^	3.3	2 ^A,C^	6.7
	Stabbing	5	16.7	4	13.3	1	3.3	0	0.0
	Other types of pain	1	3.3	2	6.7	0	0.0	2	6.7
Chi: 80.19; fd: 24; *p*-value: 0.000									
T5 **	No pain	6 ^A^	20.0	7 ^C^	23.3	19 ^C^	63.3	3 ^A^	10.0
	Acute	2 ^A^	6.7	8 ^A,D^	26.7	4 ^A,D^	13.3	12 ^D^	40.0
	Dull	1 ^B,C^	3.3	7 ^B^	23.3	0 ^C^	0.0	0 ^C^	0.0
	Sensitive	8	26.7	3	10.0	5	16.7	11	36.7
	Throbbing	9 ^A^	30.0	0 ^C^	0.0	1 ^C^	3.3	2 ^A,C^	6.7
	Stabbing	3	10.0	1	3.3	1	3.3	0	0.0
	Other types of pain	1	3.3	4	13.3	0	0.0	2	6.7
Chi: 79.99; fd: 24; *p*-value: 0.000									
T6 **	No pain	11 ^A^	36.7	8 ^A^	26.7	25 ^C^	83.3	10 ^A^	33.3
	Acute	3	10.0	8	26.7	2	6.7	6	20.0
	Dull	0	0.0	6	20.0	0	0.0	0	0.0
	Sensitive	6 ^B,D^	20.0	2 ^B^	6.7	2 ^B^	6.7	11 ^D^	36.7
	Throbbing	4	13.3	1	3.3	0	0.0	1	3.3
	Stabbing	2	6.7	2	6.7	1	3.3	1	3.3
	Other types of pain	4	13.3	3	10.0	0	0.0	1	3.3
Chi: 59.16; fd: 21; *p*-value: 0.000									
T7 **	No pain	14 ^A^	46.7	12 ^A^	40.0	27 ^C^	90.0	13 ^A^	43.3
	Acute	4	13.3	7	23.3	1	3.3	6	20.0
	Dull	0	0.0	4	13.3	0	0.0	0	0.0
	Sensitive	6 ^C,D^	20.0	1 ^C^	3.3	1 ^C^	3.3	9 ^D^	30.0
	Throbbing	4	13.3	1	3.3	0	0.0	1	3.3
	Stabbing	1	3.3	1	3.3	0	0.0	0	0.0
	Other types of pain	1	3.3	4	13.3	1	3.3	1	3.3
Chi: 50.36; fd: 21; *p*-value: 0.000									

** Statistically significant results (*p* < 0.01). Different superscript letters in the rows indicate in which groups the significant differences occurred with Bonferroni’s post hoc tests. ^A^ = *p* < 0.01 vs. CON; ^B^ = *p<* 0.01 vs. LF; ^C^ = *p* < 0.01 vs. LO; ^D^ = *p<* 0.01 vs. INV.

## Data Availability

The data presented in this study are available on request from the corresponding author. The data are not publicly available due to privacy and ethical issues.
